# Perceptions and Reintegration Experiences of Albanian Health Care Staff Returning from Abroad

**DOI:** 10.3390/ijerph22071014

**Published:** 2025-06-26

**Authors:** Vasilika Prifti, Aurela Saliaj, Sonila Qirko, Emirjona Kicaj, Rudina Çerçizaj, Juljana Xhindoli, Liliana Marcela Rogozea

**Affiliations:** 1 Faculty of Medicine, Transilvania University of Brasov, 500036 Brasov, Romania; sonila.qirko@unitbv.ro (S.Q.); emirjona.kicaj@unitbv.ro (E.K.); rudina.cercizaj@unitbv.ro (R.Ç.); r_liliana@unitbv.ro (L.M.R.); 2 Faculty of Health, University of Vlora, 9401 Vlora, Albania; aurela.dai@univlora.edu.al (A.S.); juljana.xhindoli@univlora.edu.al (J.X.)

**Keywords:** migration, health care personnel, reasons for returning, contribution, challenges

## Abstract

International healthcare staff migration remains a persistent issue, particularly for low- and middle-income countries facing health workforce shortages. While motivations for migration have been well-studied, limited attention has been given to the experiences of healthcare professionals returning to their countries of origin. This study explores the perceptions and reintegration experiences of healthcare workers who returned to Albania after working abroad. A mixed-methods approach was employed. Data collection was conducted during January and February 2025. Quantitative data were collected from 24 healthcare professionals using structured questionnaires, while qualitative insights were gathered through semi-structured interviews. Thematic analysis, following Braun and Clarke’s framework, was used for qualitative data interpretation. The main reasons for return included family-related motivations (41.7%) and professional challenges abroad (33.3%). Over half of participants (54.2%) found work immediately upon return, while others experienced difficulty re-entering the workforce or worked outside their profession. Most returnees (91.7%) believed they were contributing positively to the healthcare system. Challenges included delayed employment, low wages, inadequate infrastructure, and bureaucratic obstacles. Despite improvements, perceptions of the healthcare workforce in Albania remained mixed. Returning healthcare professionals offer valuable skills gained abroad but face reintegration barriers. Policies recognizing foreign qualifications, offering employment support, and opportunities for returnees are critical to optimize their contribution to the national health system.

## 1. Introduction

This study provides insights into returnee healthcare professionals’ experiences that can inform reforms in professional development policy, reintegration strategies, and support systems at the national level. Nursing migration is not a recent phenomenon. It has occurred throughout history and will continue to occur as long as healthcare staff is required and as long as globalization continues. Various global meanings and processes regarding healthcare professionals’ global movement place a significant proportion of healthcare workers migrating for work. Factors that drive nurses to seek employment elsewhere are, most notably, the search for better wages and good job prospects [1]. The search for new challenges, career development, better qualifications, and a broader view of the world’s cultures and languages are also typical reasons for the pursuit of a nursing profession in another country [2]. There are both—more and less severe—consequences of this migration phenomenon. The significant shortage, for example, of healthcare workers affects the quality of healthcare provision, leading to prolonged procedures, increased waiting periods, delays in receiving treatment, and the decline in the overall quality of healthcare provision [2]. On the other hand, the employing healthcare provider of the host country does not try to offer decent work, suitable work and rest conditions, fair pay, reasonable professional and development opportunities, the nurse may decide to quit and go back to the country of origin [3]. Global patterns of international migration of health care professionals, particularly nurses and nursing staff, are part of broader professional mobility trends that are increasing in volume and diversity. Less developed regions experience the smallest migration volumes. The number of departures from those regions is believed to be significant due to high levels of emigration restrictions, including quota systems, limiting professional growth, financial constraints, and lack of continuing education opportunities. In this context, inevitable migration of nursing staff is regarded as professional investment with a higher return that could not have been reached if the staff did not work abroad [3,4]. Nursing workforce issues and implications need to be understood at three interconnected levels: macro-global trends of nursing workforce development according to nurse/midwife to population density, migration of the nursing workforce, and nurse migration policy enforcement. There is a prevalent, albeit controversial, notion that nursing staff migration reaps wealth and advantages for many stakeholders. However, home countries of push factors workforce loss are often concerned about the benefits of migration.

While the World Health Organization (WHO) first began discussing the migration of healthcare workers, specifically the loss of staff from developing countries to richer ones, in the late 1990s, interest in the issue has only accelerated over the past decade [5]. There is now a significant body of literature demonstrating both the factors that push nurses to migrate and “pull” them to other countries [6]. However, the focus on migrant healthcare staff has been largely restricted to understanding why and where people leave as opposed to what happens when those who migrate wish to, or are forced to, come back. Because of this, there is now an emerging field of research examining the reintegration of healthcare professionals, although it is less well-developed [6].

As early as 1978, Mejia emphasized the global implications of nurse and physician migration, highlighting its destabilizing effects on health systems in source countries [7]. Recent analyses by James Buchan and colleagues have reinforced this view, calling for sustainable workforce planning and ethical recruitment [8]. Additionally, the WHO’s Health Workforce Support and Safeguards List (2023) and the European Observatory’s regional policy briefs underline the importance of coordinated return strategies for migrant health workers [6].

The migration of nurses presents complex challenges. Source countries, especially in Eastern and Southeastern Europe, experience workforce shortages, longer wait times for services, and declining healthcare quality due to the outflow of trained professionals. Furthermore, the cost of education and training for nurses who later emigrate is a significant loss of public investment [9]. On the other hand, destination countries benefit from a more diverse and often highly skilled nursing workforce, especially in systems under strain due to staff deficits or demographic shifts [10]. As a result, healthcare professionals moving between countries may have wildly divergent experiences and potentially perceive re-entry into the workforce differently due to this. After their return, changes in perceptions, applications, difficulties in the adaptation phase and differences in the education they receive attract attention. A growing area of concern—and one less developed in the literature—is the reintegration of healthcare professionals who return to their home countries after working abroad. These returnees often face issues related to professional recognition, bureaucratic employment processes, and misalignment between their acquired skills and the needs or expectations of the local system [11]. Studies from countries like Croatia, Romania, and Latvia show that returning healthcare workers frequently encounter institutional inertia, identity challenges, and psychosocial stress due to unmet expectations and limited reintegration support [12]. This research aims to explore the perception of healthcare staff returning from work abroad to understand the issues they face, the problems, and the discussions on this issue.

## 2. Materials and Methods

### 2.1. Study Design

Although some demographic variables were summarized numerically, the study design is primarily qualitative. These descriptive elements were intended to support the qualitative exploration rather than to conduct inferential statistical analysis. This study employed a mixed-methods design to explore the perceptions and reintegration experiences of healthcare professionals who returned to Albania after working abroad. The combination of quantitative and qualitative approaches allowed for both measurable trends and deeper insights into individual experiences. The qualitative character of the work required an interview-based design, as the aim is to provide in-depth understanding and a personal insight into information that cannot be reached by simple observations or analyses of documents. To extract a persons’ thoughts and feelings and thus understanding their perception or attribution, it was necessary to directly give them the opportunity to speak out. Interviews were recognized as an appropriate method since they can gather full and open accounts, with the great advantage of also being less restricted by the interviewer’s presence than other methods might be.

### 2.2. Study Setting

The study was conducted in the southern region of Albania, mainly health care staff that work in Vlora hospital, health care centers and Fier hospital during the months of January and February 2025.

### 2.3. Participants

Participants were recruited using convenience sampling based on accessibility and willingness. Recruitment was done through direct invitation at the hospitals where the study was authorized. The hospitals included were chosen based on their regional importance and accessibility during the study period.

A total of 24 participants were recruited using a convenience sampling technique, based on their accessibility and willingness to participate. All participants were healthcare professionals, though the sample was predominantly nurses who had previously worked abroad and returned to Albania. Most participants were nurses with varying durations of professional experience outside the country. Upon their return, they were employed in a range of healthcare settings, including regional hospitals, primary healthcare centers, private medical institutions, universities, and administrative roles. The group represented a diversity of experiences related to migration, reintegration into the local health system, and adaptation to professional environments in Albania. The inclusion criteria required that participants had spent a minimum of one year working in a foreign healthcare system and were residing in Albania at the time of data collection.

### 2.4. Variables

The study collected quantitative data on participants’ age group, country of return, years of work experience abroad, and time since returning to Albania. Additional variables included years of local work experience, employment status upon return, and type of institution employed in (e.g., public hospital, primary care, private sector). Participants were also asked whether they felt they were contributing to the healthcare system using knowledge gained abroad. Open-ended responses regarding reintegration challenges and perceptions of the current workforce were categorized thematically for analysis.

### 2.5. Data Collection

Thematic analysis followed Braun and Clarke’s six-phase framework for qualitative data interpretation. These steps include familiarization, coding, theme generation, reviewing themes, defining and naming themes, and writing up. Integration of qualitative and quantitative strands followed best practices in mixed-methods research as recommended by Creswell and Plano Clark in their study published in 2018 [13].

The following guiding questions were used in the semi-structured interviews: 1. What were your main reasons for deciding to return to Albania after working abroad? 2. How was your experience reintegrating into the Albanian healthcare system? 3. Did you face any challenges when seeking employment upon return? If so, what were they? 4. How do you perceive your contribution to the healthcare system since your return? 5. Do you feel your qualifications and international experience are recognized or valued locally? 6. How do you compare the working conditions here to those you experienced abroad? 7. What support (if any) did you receive upon your return? 8. How has your return affected your personal and professional life? 9. What changes would you recommend to improve the reintegration process for returning healthcare professionals? 10. Do language or cultural differences affect your reintegration? How so?

Interviews were conducted face-to-face and were based on a semi-structured guide. Responses were recorded in Google Forms with additional field notes. Interviews were not audio-recorded. Data collection was conducted by the principal investigators. Guiding questions focused on motivations for return, professional challenges, adaptation experiences, and perceived impact on the healthcare system.

Quantitative data were collected using a structured questionnaire, which gathered information on demographic characteristics, years of work abroad, country of return, time since return, employment status, and workplace type. Qualitative data for this research was collected using semi-structured interviews. These were face-to-face so as to allow open-ended responses, which could be probed for more information or clarification if necessary, to try and capture the nuance of people’s experiences in their own words. Interviews also provided an opportunity to record researchers’ observations of participants, which have important implications for interpretation. The semi-structured interviews were sent to google forms and comments were immediately noted to add texture and contextualization.

### 2.6. Bias

To minimize interviewer bias, the interviews were conducted using a semi-structured guide to ensure consistency across participants, and interviewers were trained to maintain a neutral stance. All interviews were sent to goggle forms and reviewed to ensure accuracy and reliability of interpretation. Despite these measures, the subjective nature of qualitative inquiry means that researcher interpretation may still be influenced by contextual and interpersonal factors.

### 2.7. Study Size

Sample size was determined based on the principle of data saturation—when no new themes emerged during subsequent interviews. As this was an exploratory mixed-methods study, the sample size was not determined through power calculations but guided by the aim to capture in-depth perspectives of returning healthcare professionals [14]. A total of 24 participants were included, which was considered sufficient to reach thematic saturation for the qualitative component, in line with accepted practices for studies of this nature. 

### 2.8. Quantitative Variables

Age Group: Participants in the study were distributed across three age categories: 25–34 years, 35–44 years, and 45–54 years.

Years of Local Experience: This variable reflects the number of years participants have worked within the Albanian healthcare system.

Time Since Returning from Emigration: Participants reported varying durations since their return to Albania, categorized as less than 1 year, 1–5 years, or more than 5 years.

### 2.9. Statistical Methods

Quantitative data were analyzed using IBM SPSS Statistics for Windows, Version 25.0 (IBM Corp., Released 2012, Armonk, NY, USA). Descriptive statistics were applied to summarize demographic and migration-related variables. This included frequencies and percentages for categorical variables such as employment status, country of return, time since return, and perceived contribution to the healthcare system. Means and standard deviations were calculated for continuous variables, including age and years of professional experience in Albania. Open-ended responses to structured questions were categorized thematically to allow for frequency-based interpretation of qualitative-type responses. For the qualitative component, data from semi-structured interviews were analyzed manually using thematic analysis, based on Braun and Clarke’s six-step framework. This method enabled the identification of key themes related to return motivations, reintegration experiences, and perceptions of the local healthcare workforce. Due to the exploratory design and relatively small sample size, no inferential statistical tests were conducted.

### 2.10. Ethical Considerations

Participation was voluntary, and all participants provided informed consent with the option to withdraw at any time. Anonymity and confidentiality were assured throughout the research process. Ethical approval for the study was obtained from the scientific ethics committee of University “Ismail Qemali” No. 134/9 as well as written approval to conduct the research was also obtained from local health authorities in Vlora municipality on 11 July 2024.

## 3. Results

The demographic and professional characteristics of the study participants are shown in Table 1. The mean age of participants is 39.1 ± 9.7. The majority of participants were female (66.7%) and aged between 35 and 44 years (45.8%). Among marital status categories, the majority of participants were married (66.7%). The predominant socio-professional group was nurses, accounting for 91.7% of participants.

The demographic and migration backgrounds of the returning healthcare professionals are summarized in Table 2. Participants varied in terms of years spent abroad, countries of return, and the duration since their return, reflecting a diverse range of migration experiences.

### 3.1. Motivations for Return

Out of 24 participants, the majority (41.7%) cited family-related reasons as the primary motivation for returning to their home country. Professional challenges, including non-recognition of qualifications and workplace dissatisfaction, accounted for 33.3% of the responses. Health-related concerns and administrative/documentation issues were each mentioned by 12.5% of participants. A smaller proportion expressed a desire to contribute to their home country (8.3%) or faced difficulties adapting linguistically and culturally (4.2%).

### 3.2. Employment Reintegration

Among 24 participants, 54.2% reported that they found a job immediately after returning, indicating relatively smooth re-entry into the workforce for more than half of the respondents. However, a significant portion (33.3%) stated that they found employment, but only after facing considerable difficulty, suggesting that reintegration into the job market was challenging for many.

Additionally, 8.3% of participants were able to secure work, but not within their professional field, which may reflect issues related to professional recognition or limited opportunities in their area of expertise. Only 4.2% of respondents were still actively searching for a job at the time of the survey, highlighting that while most had reintegrated, a small minority continued to face unemployment. This distribution suggests that although the majority managed to re-enter the labor market relatively quickly, a notable proportion encountered professional or systemic barriers that complicated their transition. Following their return, participants were employed across a range of institutions, with the Regional Hospital of Vlora employing the highest proportion (25%). A significant number also found positions in other public hospitals (20.8%), primary care centers (12.5%), and the private health sector (16.7%). A smaller proportion engaged in academic roles or administrative positions, while 4.2% remained unemployed at the time of data collection. These findings reflect a relatively diverse redistribution of the returning workforce across both public and private health sectors, with a notable concentration in regional hospital services.

### 3.3. Perceived Contribution and System Improvement

#### Qualitative Themes

Thematic analysis revealed three central themes: (1) systemic reintegration challenges, (2) psychosocial adjustment, and (3) professional disillusionment. For instance, one nurse stated, “Returning felt like starting over. I had to prove myself again, despite having ten years of experience abroad”. Another participant described, “The bureaucracy was worse than expected. I was sent to a remote area without support”. Others emphasized the emotional toll: “I missed my country, but coming back was much harder than I imagined”. These themes reflect the nuanced, often conflicted, experiences of returning professionals.

As shown by the responses in this section, most returning healthcare professionals (91.7%) felt they were able to contribute meaningfully to their home country through the skills and knowledge acquired abroad. This suggests a strong sense of professional reintegration and the potential value of return migration in strengthening the local health system. However, a small proportion (8.3%) did not perceive such a contribution, highlighting the need for more inclusive policies or environments that better leverage returning professionals’ international experience.

### 3.4. Challenges upon Return

Participants described a wide range of challenges encountered after returning to their home country. The most common issues were related to employment—including delayed hiring, assignments in remote locations, and limited opportunities for professional development—mentioned by 28.6% of respondents. Another 19.0% cited workplace difficulties, such as low salaries, lack of equipment, and understaffed facilities. Bureaucratic delays and the need for professional reintegration were also frequently mentioned. A smaller portion reported struggles with social adaptation, family-related issues, or stated they faced no challenges.

Participants expressed a range of opinions regarding the current state of healthcare personnel. 25% acknowledged noticeable improvements in working conditions, training, and professionalism compared to previous years. Similarly, 16.7% highlighted the competence and dedication of the current staff, referring to them as motivated and well-prepared. On the other hand, 29.2% described the working conditions as difficult, citing issues like outdated or insufficient medical equipment, overwhelming workloads, and general infrastructure challenges. Another 16.7% focused on financial dissatisfaction, particularly low wages and the need for multiple jobs to sustain themselves. A small number (8.3%) offered more critical or pessimistic views, mentioning poor ethics or unprepared personnel, while one participant (4.2%) stated no opinion. These responses illustrate a mixed perception of the current healthcare workforce in Albania—balancing progress and professional strength with persisting structural and financial challenges.

## 4. Discussion

The health systems in many low and middle-income countries have been impacted by a severe shortage of human resources for health, including the migration of health personnel. This issue has led many health professionals in low-income countries to work abroad. Exploring the perception of nursing staff returning from work abroad is essential, given increasing changes in international nurse migration. In narrating their experiences, the voices of the nursing staff are acknowledged upon returning from work abroad, as well as their perception, attitude, and what they can achieve in their own country, thus contributing to the filling the gap within the literature [15]. Nursing staff who have worked abroad and returned to their own country are expected to better understand, adapt, and contribute to improving the health care system in their country. As such, exploring the willingness of nursing staff to work or contribute in their own country after gaining experience working abroad becomes significant.

This study aimed to explore the experiences and perceptions of healthcare professionals returning to Albania after working abroad, shedding light on the multifaceted process of reintegration into the local healthcare system. The findings revealed five major themes: motivations for return, employment reintegration, reintegration challenges, perceived contributions, and perspectives on the healthcare workforce. Although the issue of healthcare worker migration has been widely studied globally, research focusing specifically on the Albanian context remains limited. Notable contributions include a recent survey-based study, which investigated the motivations and future intentions of doctors and nurses in Albania regarding emigration [16]. The findings revealed that a significant proportion of healthcare professionals are actively seeking opportunities abroad, primarily due to dissatisfaction with local working conditions, low pay, and limited career advancement prospects. Similarly, Gëdeshi et al. in 2024 examined the emigration of medical doctors from Albania and found that the majority had no intention of returning, pointing to a broader pattern of permanent professional migration and concerns over a potential “brain drain” within the Albanian healthcare system [17].

While these studies provide important insights into the push factors driving emigration, they do not extensively address the experiences of returning healthcare professionals—a critical yet underexplored aspect of the migration cycle. In this context, the present study contributes to filling this gap by focusing specifically on the reintegration experiences, perceptions, and challenges of Albanian healthcare staff who have returned after working abroad. By doing so, it adds a new perspective to the national discourse on migration and health workforce sustainability.

Participants most frequently cited family-related reasons—such as being separated from loved ones or the desire to raise children in their home culture—as the main motivation for return. This mirrors findings who emphasized the emotional and social factors behind return migration, especially among women healthcare workers in Romania [18]. Alongside family motives, participants also returned due to professional dissatisfaction abroad, including diploma non-recognition, racism, and poor working conditions. These findings align with a research who found that healthcare professionals in Greece and Bulgaria often faced systemic barriers such as credentialing issues and limited promotion opportunities abroad [19].

Although more than half of the participants found work shortly after returning, many described bureaucratic delays, poor placement processes, and non-meritocratic hiring mechanisms. Some were initially appointed to remote areas, often far from their families and without travel cost reimbursement. This is consistent with studies in Serbia and North Macedonia, where returnees reported similar frustrations with untransparent hiring practices and limited recognition of their international experience [20,21,22]. Participants also noted a lack of clear institutional support for their reintegration, reinforcing the conclusions of WHO (2020) [23], which emphasized the absence of structured returnee reintegration policies in many low- and middle-income countries. The mismatch between the expectations of returnees and the reality of local healthcare systems often leads to underutilization of skills, job dissatisfaction, and in some cases, renewed consideration of emigration [24].

The study revealed a range of challenges upon return. These included financial dissatisfaction, lack of necessary equipment, heavy workloads, and limited opportunities for professional advancement. This reflects broader issues identified across the Western Balkans, where returnees often experience a deterioration in work conditions compared to those abroad [25]. Moreover, some participants reported poor working culture, including weak team collaboration, limited guidance for new staff, and in some cases, lack of ethics and professionalism among peers—issues that may stem from deeper systemic problems in Albania’s healthcare management. “I struggled to adapt to the medical terminology again. Albanian wasn’t the main language I used in my last 8 years abroad”.

These findings are important when contextualized with Eurohealth published in 2018 [26], which reports similar dissatisfaction in post-transition economies where healthcare reforms often remain incomplete, leading to infrastructure and human resource deficiencies that hamper workforce morale and effectiveness. Despite the barriers, most participants felt that they were making a meaningful contribution to the healthcare system, particularly through the application of knowledge and professional standards learned abroad. This optimism was echoed in studies from Croatia and Slovenia, where returning nurses emphasized their role in improving service delivery through the transfer of knowledge, ethics, and patient-centered care practices acquired overseas [27]. However, the lack of institutional recognition of their added value was also noted by some respondents. Without official support mechanisms, structured mentorship roles, or dedicated pathways for returnees, their potential contributions risk being underleveraged. This reflects WHO (2020) [23] findings, which stress that without formal reintegration policies, health systems miss the opportunity to maximize the impact of returning professionals.

Participants offered diverse and often contradictory views of the Albanian healthcare workforce. Some praised improvements in training and professional ethics, particularly among younger nurses. Others criticized persistent issues such as low pay, understaffing, poor infrastructure, and lack of public respect toward healthcare workers. These mixed views align with data from the European Observatory on Health Systems and Policies, which emphasizes that while technical education standards have improved across Southeast Europe, workplace conditions and human resource management remain inconsistent [26]. The reality for many returnees is a healthcare system in transition—one that has made strides in training and modernization but continues to struggle with structural and cultural challenges that directly affect staff morale and retention.

### Limitations

As with all qualitative research using convenience sampling, this study is subject to potential selection bias. Participants were selected based on their accessibility and willingness to participate, which may limit the generalizability of the findings to the broader population of returning healthcare professionals in Albania. Furthermore, the self-reported nature of the data, collected through semi-structured interviews, introduces the possibility of response bias, as participants may have presented socially desirable answers or underreported negative experiences.

An additional limitation is the limited representation of non-nursing professional categories, as the final sample consisted predominantly of nurses. Although the study was open to all returning healthcare professionals, the small number of participants from other professions restricts the broader applicability of the findings across all healthcare sectors.

## 5. Conclusions

This study provides valuable insights into the experiences of returning healthcare professionals in Albania, a group often underrepresented in migration literature. While the sample was predominantly composed of nurses, the findings underscore systemic challenges relevant across the healthcare workforce. Return migration was primarily driven by family ties and dissatisfaction with working conditions abroad. Upon return, participants faced challenges related to employment, bureaucratic hurdles, lack of professional recognition, and underdeveloped infrastructure.

Despite these obstacles, most returnees believed they were making a positive contribution to the healthcare system, particularly by applying international standards, patient-centered approaches, and technical skills acquired abroad. However, their full potential remains underutilized due to the absence of formal reintegration frameworks, limited institutional recognition, and ongoing structural weaknesses within the Albanian health sector.

To address these gaps, it is essential for policymakers to develop targeted reintegration strategies, including:

- Streamlined recognition of foreign qualifications to ensure returning professionals are employed at their appropriate skill level;
- Transparent and merit-based hiring processes to reduce arbitrary placements;
- Incentives for working in underserved regions, helping address geographic disparities in care delivery;
- Structured mentorship and leadership roles for returnees to foster professional development and system-wide learning;
- Curriculum integration of global health experiences and reintegration challenges into nursing and medical education to prepare future professionals for circular migration dynamics.

Moreover, investing in improved working conditions, equitable salaries, continuing education, and healthcare infrastructure is critical not only for retaining returnees but also for supporting the broader health workforce. Without such reforms, Albania—and other countries in similar socioeconomic contexts—risks further erosion of human capital and the loss of valuable experience brought back by internationally trained professionals.

To address these challenges, it is essential that policymakers develop targeted strategies to support the effective reintegration of returning healthcare professionals. A key priority should be the streamlined recognition of foreign qualifications, ensuring that skills and experience acquired abroad are formally acknowledged within the national health system. Additionally, the implementation of transparent and merit-based hiring processes is crucial to prevent arbitrary placement and to promote fairness in employment opportunities.

Offering incentives for returnees to work in underserved or remote areas can help address regional disparities in healthcare access, while also encouraging returnees to contribute where their expertise is most needed. Finally, creating structured mentorship or leadership roles for returning professionals would not only facilitate their integration but also enable them to share their international experience and strengthen the professional culture within local institutions. Furthermore, improving working conditions, raising wages, and investing in infrastructure are essential to retain both returning and domestically trained healthcare professionals. Without such reforms, Albania—and similar countries in the Balkans—risks continued loss of talent and underutilization of those who return with valuable international experience.

## Figures and Tables

**Table 1:** Demographic and Professional Characteristics of the Study Participants (N = 24).

Variables	N	%
**Gender**		
Female	16	66.7
Male	8	33.3
**Age Mean (SD)**	39.1 ± 9.7	
**Age group**		
25–34 years	7	29.2
35–44 years	11	45.8
45–54 years	6	25.0
**Marital status**		
Single	5	20.8
Married	16	66.7
Divorced	2	8.3
Cohabiting	1	4.2
**Profession**		
Nurse	22	91.7
Midwife-nurse	1	4.2
Physician	1	4.2
**Years of local experience Mean (SD)**	6.0 ± 5.8	1–26

**Table 2:** Migration Characteristics of Returning Healthcare Professionals (N = 24).

Variables	N	%
**Years Working Abroad**		
1–3 years	14	58.3
4–7 years	10	41.7
**Country Returned From**		
Germany	7	29.2
Greece	5	20.8
Italy	9	37.5
USA	3	12.5
**Years Working Abroad**		
1–3 years	3	12.5
4–7 years	11	45.8
**Time Since Returning**	10	41.7
<1 year	3	12.5
1–5 years	11	45.8
>5 years	10	41.7

## Data Availability

Data are contained within the article.

## Public Involvement Statement

No public involvement in any aspect of this research.

## Guidelines and Standards Statement

This manuscript was drafted against the STROBE guidelines for observational research. A complete list of reporting guidelines can be accessed via the equator network: https://www.strobe-statement.org/.
